# The Role of the Gut Microbiome in the Relationship Between the Mediterranean Diet and Cardiovascular Disease Risk

**DOI:** 10.1007/s11883-026-01463-7

**Published:** 2026-09-11

**Authors:** Adrián Hernández-Cacho, Jiaqi Ni, Jesús F. García-Gavilán, Josué Alberto Pérez-Acosta, Héctor Vázquez-Lorente, Julio Plaza-Diaz, Jordi Salas-Salvadó

**Affiliations:** 1 Departament de Bioquímica i Biotecnologia, Alimentació, Nutrició, Desenvolupament i Salut Mental ANUT-DSM, Unitat de Nutrició, Facultat de Medicina i Ciències de la Salut, Universitat Rovira i Virgili, C/Sant Llorenç, 21, Reus, 43201 Spain; 2 Institut de Recerca Biomèdica Catalunya Sud, Hospital Universitari Sant Joan de Reus, Reus, Spain; 3 Centro de Investigación Biomédica en Red Fisiopatología de la Obesidad y la Nutrición (CIBEROBN), Instituto de Salud Carlos III, Madrid, Spain

**Keywords:** Mediterranean diet, Gut microbiome, Cardiovascular disease, Metabolomics, Precision nutrition, Short-chain fatty acids

## Abstract

**Purpose of review:**

This review critically summarizes evidence linking the Mediterranean diet, gut microbiome, microbial metabolites, and cardiovascular disease risk, with emphasis on the shift from microbial taxonomy to functional pathways, multiomic integration, and translation to precision nutrition.

**Recent findings:**

Mediterranean diet adherence is associated with enrichment of fiber-degrading and short-chain fatty acid-producing taxa, greater microbial capacity for polysaccharide degradation, and more favorable bile acid, phenolic, lipid, and inflammatory profiles. Evidence from randomized trials and prospective cohort studies suggests that microbiome remodeling may partly mediate or modify the effects of the Mediterranean diet on adiposity, insulin resistance, lipid metabolism, inflammation, and cardiometabolic risk. However, effects are heterogeneous, global diversity metrics are inconsistent, and most studies rely on surrogate outcomes rather than incident cardiovascular events.

**Summary:**

The gut microbiome is a plausible mediator and modifier of Mediterranean diet-related cardiovascular protection, but it is not yet a validated clinical target. Future research should prioritize longitudinal multiomic studies with repeated measures, standardized analytical pipelines, externally validated biomarkers, and pragmatic trials testing whether microbiome-informed Mediterranean diet counseling improves adherence, cardiometabolic profiles, and cardiovascular outcomes.

**Graphical Abstract:**

**Figure:**
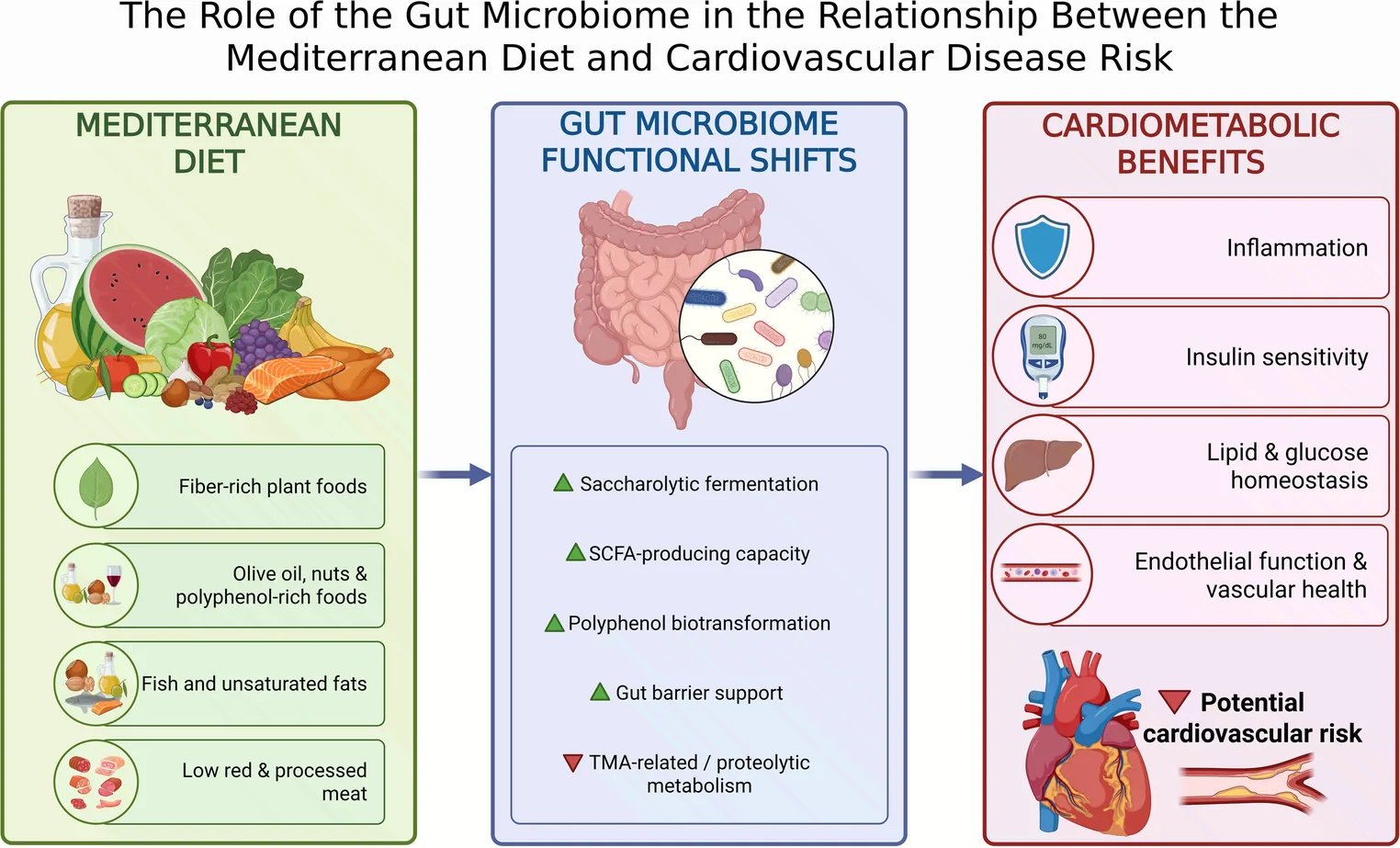

## Introduction

Cardiovascular diseases (CVDs) remain the leading cause of death worldwide despite substantial advances in their prevention and treatment, imposing an enormous burden in terms of morbidity, hospitalizations, and healthcare costs [1]. Although genetic predisposition contributes to disease susceptibility, modifiable lifestyle factors, including physical inactivity, obesity, smoking, excessive alcohol consumption, psychological stress, and unhealthy dietary habits, are major determinants of the development and progression of CVD [2]. Among these, diet is one of the most important modifiable determinants of cardiovascular health, and, of the dietary patterns evaluated, the Mediterranean diet (MedDiet) has accrued strong evidence for preventing CVD and other chronic diseases [3].

The traditional MedDiet is characterized by high consumption of fruits, vegetables, legumes, nuts, whole grains, fish, and olive oil as the principal fat source; moderate consumption of poultry, eggs, and dairy products; low consumption of red and processed meats, and moderate wine intake during meals. Given these characteristics, the MedDiet has been proposed as a transferable and sustainable dietary model for non-Mediterranean populations with a high burden of CVD [4].

A large body of observational evidence has consistently shown that greater adherence to the MedDiet is associated with a lower incidence of type 2 diabetes and major cardiovascular events and with lower cardiovascular mortality [5, 6]. These findings have since been reinforced by large prospective cohort studies with extensive follow-up and randomized controlled trials (RCTs) [6, 7]. Although evidence from RCTs with hard cardiovascular endpoints has historically been more limited, several major trials have provided robust support for its protective effects. In primary prevention, the PREDIMED trial showed that participants assigned to a MedDiet supplemented with extra-virgin olive oil or nuts experienced a lower incidence of major cardiovascular events (stroke, myocardial infarction, and cardiovascular death) compared with those assigned to a low-fat diet [8]. In secondary prevention, the CORDIOPREV study reported a 27% reduction in the risk of major cardiovascular events among individuals with established CVD assigned to a MedDiet compared with a low-fat diet [9]. More recent analyses from the same trial further showed an inverse association between the degree of adherence to the assigned dietary patterns and the incidence of recurrent major cardiovascular events during 7 years of follow-up [10]. Beyond event prevention, an energy-restricted MedDiet combined with physical activity promotion and behavioral support has also been shown to be effective in achieving clinically meaningful weight loss and improving cardiovascular risk factors among older adults with overweight or obesity and metabolic syndrome, as demonstrated in the PREDIMED-Plus trial [11]. Collectively, evidence from prospective studies, RCTs, umbrella reviews and recent meta-analyses indicates that greater adherence to the MedDiet is associated with lower risks of fatal and non-fatal cardiovascular outcomes, including among individuals with established CVD [7, 12, 13].

Despite this compelling body of epidemiological and clinical evidence, the biological mechanisms underlying the cardiovascular benefits of the MedDiet, and the marked inter-individual variability in response, remain incompletely understood. This dietary pattern provides abundant dietary fiber, monounsaturated fatty acids, vitamins, minerals, and a wide range of bioactive compounds with antioxidant and anti-inflammatory properties, all of which may contribute to cardiovascular protection [14]. More recently, growing attention has focused on the gut microbiota as a potential mediator of these effects. The MedDiet may promote favorable shifts in microbial composition and metabolite output that influence host cardiometabolic pathways through the gut–heart axis [15, 16]. These interactions may explain part of the cardiovascular benefits of the MedDiet while also contributing to the heterogeneity of individual responses.

This review critically summarizes current evidence on the interactions between the MedDiet, the gut microbiome, microbial metabolites, and cardiovascular disease. We discuss the mechanistic pathways linking diet-induced microbial changes to cardiovascular health, identify the principal translational challenges that currently limit clinical implementation, and highlight future opportunities for microbiome-based precision nutrition strategies in CVD prevention and management. The added value of this narrative review lies in integrating evidence across microbial composition, microbial functional pathways, metabolomics, host cardiometabolic phenotypes, and cardiovascular outcomes within a unified framework. Rather than focusing primarily on taxonomic changes, we emphasize functionally relevant microbial and host-microbial pathways and consider the gut microbiome as a potential mediator and modifier of the cardiometabolic response to the MedDiet. We further distinguish mechanistic and associative evidence from findings that have undergone sufficient validation for clinical translation, thereby linking current microbiome and multiomic research with the emerging field of microbiome-informed precision nutrition.

## Review Methodology

This narrative review was informed by a targeted literature search conducted in PubMed/MEDLINE, Scopus, and Web of Science for publications from January 2000 through July 2026. The search strategy combined terms related to the Mediterranean diet (“Mediterranean diet,” “Mediterranean dietary pattern,” “MedDiet”), the gut microbiome (“gut microbiota,” “gut microbiome,” “intestinal microbiota,” “metagenomics”), microbial and host-derived metabolites (“metabolomics,” “short-chain fatty acids,” “SCFA,” “trimethylamine N-oxide,” “TMAO,” “bile acids,” “phenylacetylglutamine,” “PAGln,” “polyphenol metabolites,” “lipopolysaccharide,” and “endotoxemia”), and cardiovascular or cardiometabolic outcomes (“cardiovascular disease,” “atherosclerosis,” “cardiometabolic risk,” “cardiovascular events,” “myocardial infarction,” “stroke,” “cardiovascular mortality,” “insulin resistance,” “lipid metabolism,” and “inflammation”).

Because the objective of this article was to provide a critical narrative synthesis rather than a systematic review or quantitative meta-analysis, studies were selected according to their relevance to the biological, clinical, and translational questions addressed in the review. Particular emphasis was placed on randomized controlled trials, prospective cohort studies, human microbiome and multiomic studies, systematic reviews and meta-analyses, while mechanistic experimental studies were considered when necessary to contextualize biological pathways for which direct human evidence remains limited. The synthesis focused particularly on evidence linking Mediterranean diet exposure with microbial composition or function, microbiome-related metabolites, cardiometabolic phenotypes, and cardiovascular outcomes.

### Functional Microbial Pathways Linking the Gut Microbiome to Cardiovascular Disease

A major conceptual shift in microbiome–cardiovascular research has been the move from taxonomic description toward understanding microbial function. Although the relative abundance of individual taxa varies across populations and according to dietary habits, medication use, geography, and sequencing platforms, microbial metabolic pathways provide more reproducible and biologically interpretable links between diet and CVD [17, 18]. The gut microbiome is thus better understood not as a list of bacteria but as a metabolic interface capable of transforming dietary substrates into bioactive compounds that influence lipid metabolism, bile acid signaling, vascular inflammation, endothelial function, platelet activation, insulin sensitivity, and immune regulation [19–21].

Among these pathways, trimethylamine-N-oxide (TMAO) has the most extensive experimental and clinical evidence base. Gut bacteria convert dietary choline, phosphatidylcholine, betaine, and L-carnitine into trimethylamine, which is oxidized in the liver to TMAO. Experimental studies suggest that this pathway may contribute to atherogenesis and thrombosis through effects on cholesterol balance, macrophage foam cell formation, vascular inflammation, endothelial dysfunction, and platelet hyperreactivity [22–26]. However, circulating TMAO should be interpreted cautiously because its concentrations are also influenced by renal function, seafood intake, host genetics, hepatic regulation, and interindividual variation in microbial TMA-producing capacity [27–30]. Moreover, Mendelian randomization and intervention studies have yielded inconsistent evidence regarding a causal role of TMAO in cardiovascular events. Therefore, TMAO is best viewed as a context-dependent host–microbial metabolite rather than a specific marker of harmful diet or microbial activity [23, 31–34].

Short-chain fatty acids (SCFAs), mainly acetate, propionate, and butyrate, represent a second major functional pathway and the one most consistently linked to adherence to the MedDiet. They are produced through bacterial fermentation of non-digestible carbohydrates and may influence cardiometabolic health through G protein-coupled receptor signaling, immune modulation, gut barrier integrity, blood pressure regulation, and histone deacetylase inhibition [35, 36]. However, most supportive data derive from animal models and fecal measurements, which poorly reflect systemic SCFA exposure. Direct human evidence linking SCFAs to hard cardiovascular events is still lacking. SCFA-related pathways are therefore best regarded as robust functional signatures of high-fiber dietary patterns and MedDiet adherence rather than proven causal mediators of cardiovascular protection [35, 36].

Bile acids provide another host–microbial signaling axis. Gut bacteria deconjugate and transform primary bile acids, modifying the intestinal bile acid pool and influencing FXR- and TGR5-related pathways involved in hepatic bile acid synthesis and regulation, lipid metabolism, glucose homeostasis, and inflammatory tone [37]. Although experimental evidence supports an important role for bile acid signaling in cardiometabolic regulation, direct evidence linking microbiome-mediated bile acid remodeling to cardiovascular outcomes in humans remains limited.

In parallel, microbial metabolism of dietary polyphenols generates smaller phenolic metabolites that may contribute to antioxidant, anti-inflammatory, and endothelial effects. These pathways are particularly relevant to the MedDiet because its plant foods, extra-virgin olive oil, nuts, legumes, fruits, vegetables, and moderate wine intake provide abundant substrates for microbial biotransformation [38].

Phenylacetylglutamine (PAGln), generated from microbial phenylalanine metabolism followed by host conjugation, has emerged as another CVD-relevant metabolite acting through adrenergic receptor signaling to promote platelet activation and thrombosis-related pathways. Its evidence base is less mature than that of TMAO, but PAGln reinforces the broader concept that microbial amino acid metabolism may contribute to atherothrombotic risk [39–41]. However, prospective interventional and genetic studies are still needed to establish its causal role in cardiovascular disease.

Finally, gut barrier dysfunction provides a complementary inflammatory mechanism. Increased intestinal permeability facilitates translocation of lipopolysaccharide and other microbial products, promoting metabolic endotoxemia, innate immune activation, and low-grade systemic inflammation. These processes are biologically interconnected with microbial metabolites, bile acid signaling, vascular function, renal function, cardiometabolic status, and medication exposure [42].

**Fig. 1:**
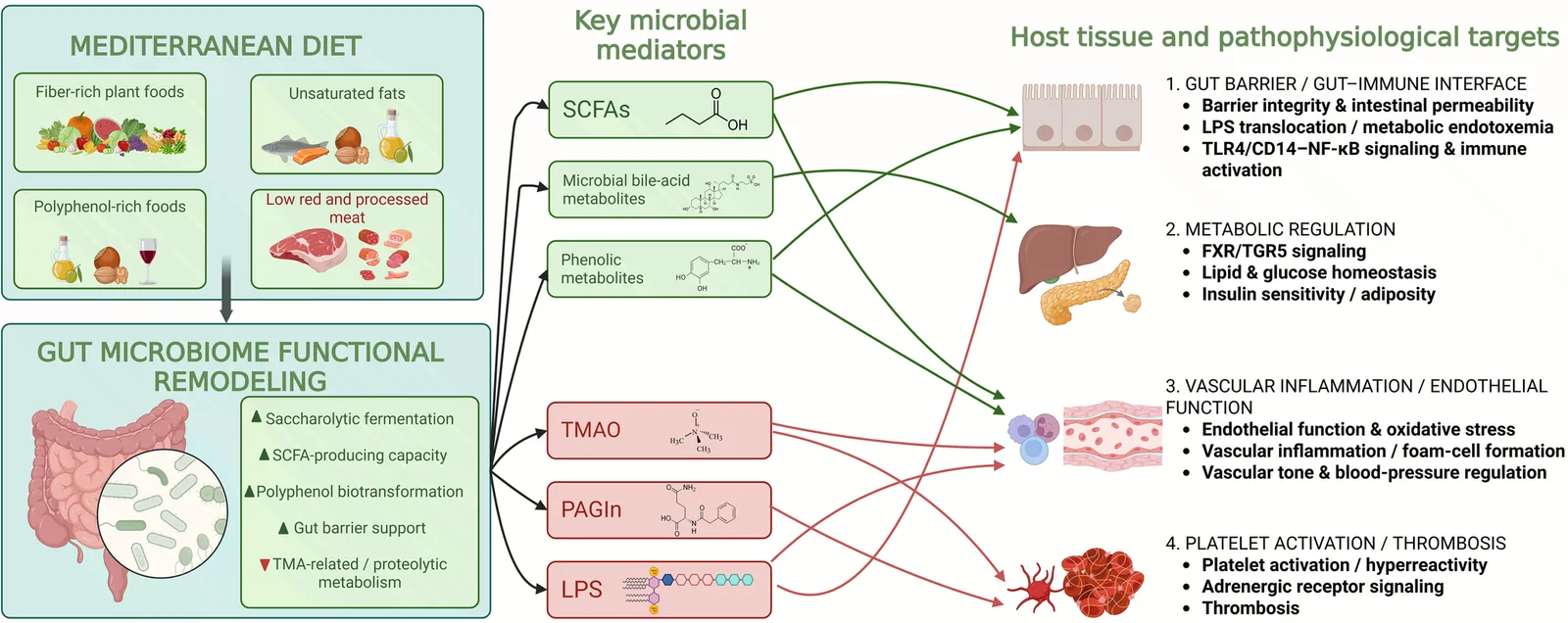
Proposed pathophysiological pathways linking the Mediterranean diet, gut microbiome functional remodeling, microbial metabolites, and cardiovascular disease risk

Taken together, current evidence indicates that microbial function is more informative than microbial composition alone for understanding how diet influences cardiovascular disease risk [43, 44]. The proposed pathophysiological pathways linking Mediterranean diet components, gut microbiome functional remodeling, microbial metabolites, and cardiovascular disease risk are summarized in Fig. 1. Table 1 summarizes these microbiota-derived metabolites and pathways implicated in atherosclerotic cardiovascular disease (ASCVD)-relevant mechanisms.

**Table 1:** Microbiota-derived metabolites and host–microbial pathways implicated in ASCVD-relevant mechanisms

Microbiota-derived metabolite	Major microbial origin/pathway	Key host targets/mechanisms	ASCVD-relevant phenotypes/evidence
Trimethylamine-N-oxide (TMAO) [23, 26–28, 31, 32, 34]	Gut microbial conversion of dietary choline, phosphatidylcholine, betaine, and L-carnitine into trimethylamine; hepatic oxidation by flavin-containing monooxygenases	Cholesterol balance; reverse cholesterol transport; macrophage foam cell formation; vascular inflammation; endothelial oxidative stress; platelet hyperreactivity and thrombosis	The strongest evidence among microbiota-derived metabolites: experimental atherosclerosis models, human cohort studies, and meta-analyses linking circulating TMAO with CVD events and mortality
Short-chain fatty acids (SCFAs): acetate, propionate, butyrate [35, 36]	Bacterial fermentation of non-digestible carbohydrates and dietary fiber	GPCR signaling; renin secretion and blood pressure regulation; immune homeostasis; gut barrier integrity; histone deacetylase inhibition, particularly for butyrate	Mechanistically linked to blood pressure regulation, inflammation, barrier function, and attenuation of hypertensive cardiovascular damage in experimental models; direct human ASCVD endpoint evidence remains limited
Secondary bile acids [37]	Microbial deconjugation and transformation of host-derived primary bile acids	Intestinal bile acid pool composition; FXR–FGF15/19 signaling; TGR5 signaling; hepatic bile acid synthesis; lipid and glucose metabolism; inflammatory tone	Relevant to cardiometabolic regulation through bile acid, lipid, and glucose homeostasis; direct causal evidence for ASCVD remains less developed than for TMAO
Microbial phenolic metabolites [38]	Microbial biotransformation of dietary polyphenols from plant-rich foods into smaller phenolic derivatives	Oxidative stress modulation; anti-inflammatory signaling; endothelial function; vascular redox balance	Associated with Mediterranean diet adherence and more favorable cardiovascular health profiles; evidence is mainly associative and biomarker-based in humans
Lipopolysaccharide (LPS)/metabolic endotoxemia [43, 44]	Translocation of Gram-negative bacterial products in the setting of impaired gut barrier integrity	TLR4/CD14-mediated innate immune activation; NF-κB signaling; systemic low-grade inflammation; endothelial activation; immune-cell recruitment	Mechanistically linked to metabolic inflammation and experimental atherosclerosis; barrier restoration has been shown to attenuate endotoxemia-induced inflammation and atherosclerotic lesions in APOE^−/−^ mice
Phenylacetylglutamine (PAGln) [39, 41]	Gut microbial phenylalanine metabolism to phenylacetic acid, followed by host conjugation with glutamine	Adrenergic receptor signaling; platelet activation; thrombosis-related pathways	Emerging CVD-specific metabolite; associated with cardiovascular risk and experimentally linked to platelet activation and thrombotic potential

ASCVD, atherosclerotic cardiovascular disease; CVD, cardiovascular disease; FXR, farnesoid X receptor; GPCR, G protein-coupled receptor; LPS, lipopolysaccharide; NF-κB, nuclear Factor kappa-light-chain-enhancer of activated B cells; PAGln, phenylacetylglutamine; SCFA, short-chain fatty acid; TMAO, trimethylamine-N-oxide; TGR5, Takeda G protein-coupled receptor 5; TLR4/CD14, Toll-like receptor 4/cluster of differentiation 14

### Mediterranean Diet–Microbiome Interactions and Cardiometabolic Pathways

#### Mediterranean Diet As a Determinant of Gut Microbiota Composition and Function

Diet is one of the most influential and modifiable determinants of gut microbiota composition and function [45–47]. Short-term dietary interventions can produce measurable shifts in microbial taxa and metabolic activity within days [45–47], whereas long-term dietary patterns shape the stability, resilience, and functional capacity of the gut ecosystem [46, 48]. Food groups, macronutrients, food processing technologies, and bioactive compounds interact with the host gut microbiome by providing substrates and conditions for fermentation, polyphenol biotransformation, bile acid remodeling, and host–microbial co-metabolism [49, 50]. Consequently, habitual diet represents one of the principal environmental factors determining the metabolic potential of the gut microbiome and its contribution to host health.

The MedDiet is particularly relevant because it combines multiple microbiota-accessible substrates within a single dietary pattern: fermentable fibers from legumes, fruits, vegetables, and whole grains; unsaturated fatty acids from extra-virgin olive oil, nuts, and fish; polyphenols from plant foods and extra-virgin olive oil; and lower intake of red and processed meat. Therefore, its effects on the gut microbiome are unlikely to depend on a single food or bacterial group, but rather on the combined capacity of the dietary pattern to promote saccharolytic fermentation, polyphenol biotransformation, gut barrier support, and lower proteolytic or TMA-related metabolism [49, 51, 52].

By contrast, Western dietary patterns, typically rich in ultra-processed foods, refined carbohydrates, saturated fats, red and processed meats, and low-fiber foods, are generally associated with reduced microbial diversity, enrichment of opportunistic or pro-inflammatory microorganisms, impaired microbial metabolic capacity, and greater production of metabolites linked to proteolytic fermentation and TMA-related pathways [50]. The principal dietary determinants of gut microbial composition and function are summarized in Table 2.

**Table 2:**
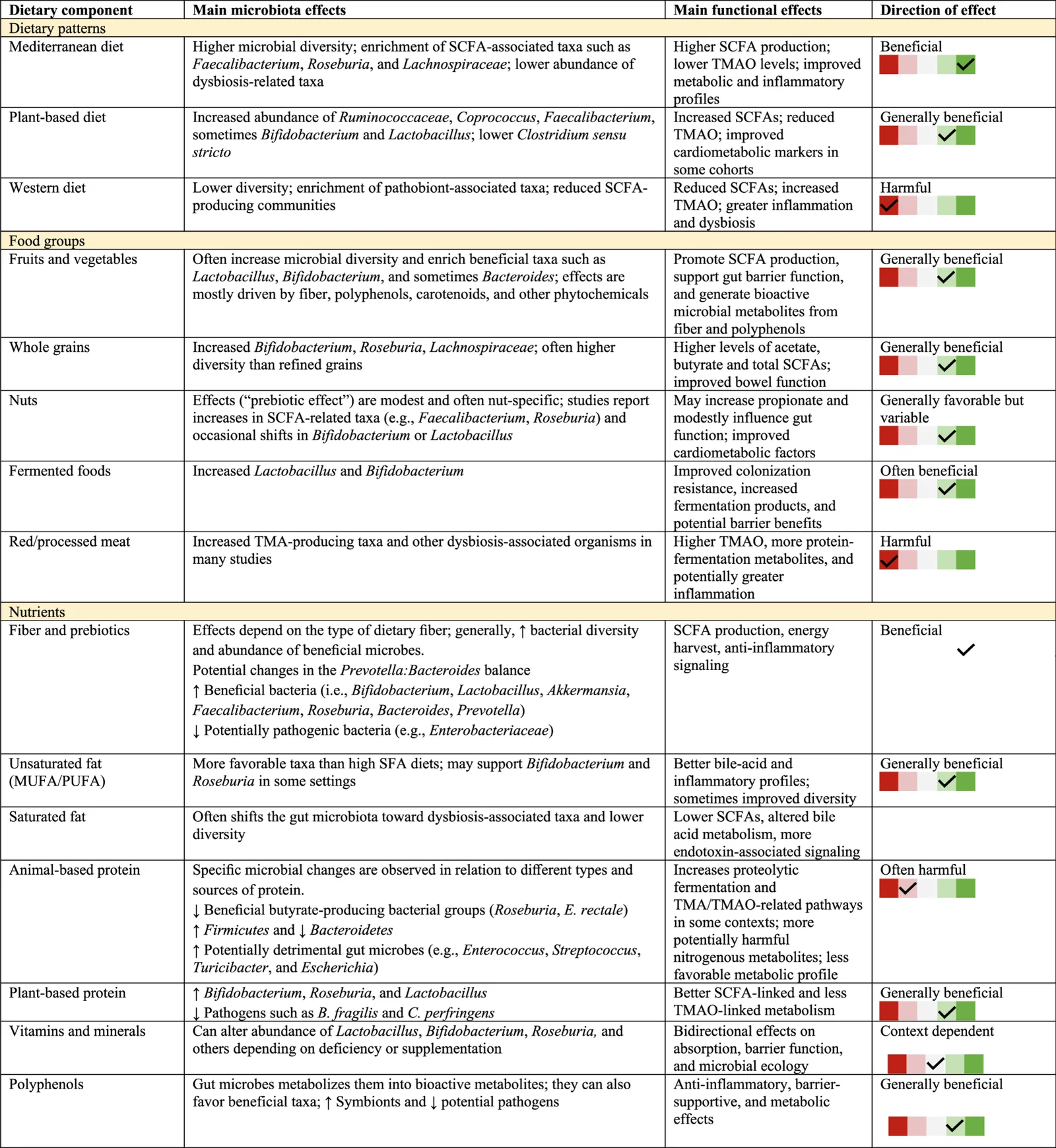
Overview of dietary patterns, food groups, and nutrients influencing gut microbiota composition and function

MUFA, monounsaturated fatty acids; PUFA, polyunsaturated fatty acids; SCFA, short-chain fatty acid; SFA, saturated fatty acids; TMAO, trimethylamine-N-oxide; ↑, increased; ↓, decreased

####  Human Evidence Linking the Mediterranean Diet, Gut Microbiome, and Cardiometabolic Health

 Human evidence linking the MedDiet, gut microbiome, and cardiometabolic health comes from randomized trials, crossover trials, and observational cohorts conducted in healthy younger and older adults, and in populations with obesity, metabolic syndrome, prediabetes, or elevated cardiovascular risk. Intervention durations have generally ranged from 8 weeks to 12 months, and most studies have used 16S rRNA gene sequencing, although an increasing number now incorporate shotgun metagenomics, metabolomics, or integrated multiomic approaches. This heterogeneity limits direct comparability but provides a useful gradient of evidence, from taxonomic shifts to functional and metabolic readouts [48, 53–68].

A central and reproducible finding across intervention studies is the enrichment of SCFA–producing taxa following MedDiet adherence. Multiple RCTs consistently report increased abundance of butyrate-producing bacteria, including *Faecalibacterium prausnitzii*, *Roseburia*, and members of the *Lachnospiraceae* and *Ruminococcaceae* families [48, 53, 55]. These compositional changes are paralleled by functional shifts toward enhanced saccharolytic fermentation, reflected in increased SCFA production and upregulation of microbial pathways involved in plant polysaccharide degradation [66, 68]. Importantly, these microbial adaptations are closely linked to improvements in host metabolic health, including enhanced insulin sensitivity, reduced postprandial glycemic responses, improved lipid profiles, and lower systemic inflammation [53, 54, 62, 63]. Conversely, MedDiet interventions appear to suppress microbial taxa and metabolic activities associated with cardiometabolic dysfunction. Several studies report reductions in pro-inflammatory or mucin-degrading species such as *Ruminococcus gnavus* and *Ruminococcus torques*, alongside decreases in potentially harmful microbial metabolites, including secondary bile acids, p-cresol, ethanol, and trimethylamine-related compounds (e.g., TMAO) [48, 53, 55, 62]. These findings suggest a shift from a proteolytic, inflammation-associated microbial profile toward a fiber-driven, metabolically favorable ecosystem. Supporting this, metabolomic analyses have shown decreased circulating levels of carnitine-derived metabolites and increased production of beneficial polyphenol-derived compounds, such as urolithins, highlighting the integration of dietary components with microbiome-mediated metabolic pathways [53, 56, 64]. In addition, recent evidence from the PREDIMED-Plus randomized clinical trial showed that a one-year lifestyle intervention based on an energy-restricted MedDiet combined with physical activity induced coordinated changes in the fecal microbiome and metabolome, including increased microbial alpha diversity, alterations in metabolic networks related to bile acids, ceramides, sphingolipids, fatty acids, and carnitine, and reductions in the abundance of the *Eubacterium hallii* group and *Dorea* [69]. Importantly, both microbiome and metabolomic changes were associated with improvements in body weight, insulin resistance, glycemic control, and other cardiovascular risk factors, providing further evidence that functional microbiome remodeling may contribute to the cardiometabolic benefits of Mediterranean lifestyle interventions.

More recent evidence has begun to extend these observations to hard cardiovascular outcomes. In the CORDIOPREV trial, greater baseline postprandial lipopolysaccharide responses were associated with a higher risk of recurrent major adverse cardiovascular events during 7 years of follow-up in patients with coronary heart disease. Both Mediterranean and low-fat dietary interventions reduced postprandial lipopolysaccharide concentrations and were associated with gut microbiota profiles linked to lower endotoxemia, while the association between postprandial endotoxemia and cardiovascular recurrence differed according to dietary intervention. These findings provide clinically relevant evidence linking gut barrier-related microbial processes with recurrent cardiovascular events, although further studies are required before postprandial endotoxemia or related microbial signatures can be considered validated tools for cardiovascular risk stratification [70].

However, not all studies report significant changes in global microbial diversity or overall community structure. Several crossover RCTs, particularly in individuals with metabolic syndrome, observed minimal differences in α- or β-diversity despite clear improvements in cardiometabolic parameters [56, 58, 61], plausibly reflecting short intervention durations, small samples, and the limited sensitivity of global diversity indices. In these studies, more subtle but biologically meaningful changes were detected at the level of specific taxa or microbial metabolites, including alterations in bile acid metabolism, acylcarnitines, and other lipid intermediates [56, 58]. These findings suggest that global diversity metrics may lack the sensitivity need to capture diet-induced microbiome modulation and underscore the importance of integrating functional and metabolomic readouts to better characterize host–microbiome interactions.

Beyond taxonomic composition, functional remodeling of the gut microbiome emerges as a key mechanism linking the MedDiet to cardiometabolic health. Shotgun metagenomic analyses demonstrate that MedDiet adherence increases microbial gene richness and promotes metabolic pathways involved in branched-chain amino acid (BCAA) degradation while reducing BCAA biosynthesis [53, 54]. Given the established association between elevated circulating BCAAs and insulin resistance, these functional changes provide a biologically plausible pathway connecting diet-induced microbiome alterations to improved metabolic outcomes. Notably, mediation analyses from large RCTs indicate that microbiome changes may account for approximately 10–20% of the beneficial effects of MedDiet interventions on adiposity and cardiovascular risk [54], supporting a partial causal role of the gut microbiome, though these estimates await external replication.

Evidence from observational studies complements these findings. Long-term adherence to the MedDiet has been associated with increased microbial capacity for plant polysaccharide degradation, higher SCFA production, and enrichment of fiber-degrading species. Inter-individual variability in baseline microbiome composition appears to modulate the metabolic response to the MedDiet [68], highlighting the potential for microbiome-informed precision nutrition strategies [71]. However, whether microbiome-guided dietary recommendations provide clinically meaningful benefits beyond conventional dietary counseling remains to be established.

Overall, current human evidence indicates that MedDiet interventions can induce potentially functionally favorable gut microbiome changes, characterized by increased fiber fermentation, enrichment of SCFA-producing taxa, and reduced generation of selected proteolytic or TMA-related metabolites. Although changes in microbial taxonomy and global diversity are less consistent across studies, functional adaptations are also observed and have been associated with improvements in insulin sensitivity, inflammation, lipid metabolism, and other cardiometabolic outcomes. It is important to consider that the magnitude of these microbiome-related benefits vary according to baseline diet, medication exposure, cardiometabolic phenotype, geography, sequencing platform, and analytical method, which likely explains much of the between-study heterogeneity. These findings support the need to move from descriptive taxonomic comparisons toward integrated microbiome–metabolome–phenotype models to better understand the contribution of the gut microbiome to Mediterranean diet–associated cardiometabolic benefits (Table 3).

**Table 3:** Evidence from human studies on Mediterranean diet, gut microbiome, and cardiometabolic health

Study, year	Study design and population	Sample size	MedDiet intervention/exposure	Microbiome assessment	Microbiome and/or metabolome findings	Cardiometabolic outcomes	Key results (Study evidence strength)
Ghosh et al., [48] (NU-AGE study)	RCT, non-frail or pre-frail European elderly adults	*N* = 612	MedDiet vs. habitual diet (12 months)	16 S rRNA gene sequencing	↑ “DietPositive”, SCFA-linked taxa (*Faecalibacterium prausnitzii*,* Roseburia*); ↓ “DietNegative” frailty-associated taxa. ↑ short- and branched-chain fatty acids; ↓ secondary bile acid/p-cresol/ethanol/carbon dioxide production.	Lower frailty and improved cognitive function; ↓ CRP and IL-17	MedDiet-induced microbiome remodeling promotes healthier aging and attenuates frailty.
Meslier et al., [53]	RCT, healthy overweight/obese participants	*N* = 82	MedDiet vs. habitual diet (8 weeks)	Shotgun metagenomics	↑ Microbial richness; ↑ SCFA-producing taxa (*Faecalibacterium prausnitzii*); ↓ proinflammatory taxa (*Ruminococcus gnavus*). ↓ Plasma and urinary carnitine levels and protein degradation products; ↑ urinary urolithins and fecal bile acid degradation.	↓ Total cholesterol, LDL and HDL cholesterol, and fecal bile acids ↑ Insulin sensitivity	Isocaloric 8-week MedDiet lowered total/LDL cholesterol and fecal bile acids and improved insulin sensitivity; benefits tracked with adherence and were mediated by increased microbial gene richness, enrichment of fiber-degrading/SCFA producing taxa (*F. prausnitzii*↑, *R. gnavus*↓) and favorable bile acid/polyphenol metabolite profiles, linking MedDiet-induced microbiome remodeling to an improved cardiometabolic risk profile.
Rinott et al., [54] (DIRECT-PLUS study)	RCT, participants with abdominal obesity/dyslipidemia	*N* = 294	Green-MedDiet vs. MedDiet vs. healthy dietary guidelines (HDG) (6 months)	16 S rRNA for all stool samples (546 stool samples); shotgun sequencing for a select subset of samples (173 samples from 90 participants)	Green-MedDiet showed graded enrichment of *Prevotella* and depletion of *Bifidobacterium* across HDG→MedDiet→Green-MedDiet; functional modules in Green-MedDiet/MedDiet favored increased BCAA-degradation pathways and reduced BCAA-biosynthesis pathways, and Green-MedDiet adherence was associated with specific taxa (e.g., *Ruminococcaceae*, *Lachnospiraceae*) and pathways (e.g., reduced cysteine biosynthesis) that correlated with greater weight loss and lower Framingham risk.	Stepwise improvements across HDG→MedDiet→Green-MedDiet in weight loss (largest in Green-MedDiet), waist circumference, blood pressure, lipid ratios (TG/HDL, TC/HDL, LDL-C/HDL-C), HOMA-IR, leptin, chemerin, GGT, and 10-year Framingham cardiovascular risk score; global microbiome change and selected taxa/pathways statistically mediated a meaningful proportion (~ 10–20%) of the Green-MedDiet effect on weight, waist circumference, and Framingham risk.	During a 6-month of weight-loss intervention, plant-enriched, polyphenol-rich Green-MedDiet and MedDiet produced graded microbiome remodeling (especially of rare taxa, with *Prevotella* ↑, *Bifidobacterium* ↓ and BCAA-degradation pathways ↑) alongside graded improvements in weight, adiposity, blood pressure, glycemic control, lipid ratios and Framingham risk; mediation analyses suggest that changes in gut microbiome composition and function partially explain the cardiometabolic benefits of the Green-MedDiet.
Muralidharan et al., [55] (PREDIMED-Plus study)	RCT, older adults with overweight/obesity and MetS	*N* = 400	Energy-reduced MedDiet + physical activity promotion (IG) vs. non-energy-reduced MedDiet (CG) (1 year)	16 S rRNA gene sequencing	No significant α/β diversity changes between groups; IG vs. CG: ↓ *Haemophilus*, ↓ *Butyricicoccus*, ↓ *Ruminiclostridium* 5, ↓ *Eubacterium hallii*, ↑ *Coprobacter*, ↑ *Ruminococcaceae NK4A214*; both groups: ↑ *Lachnospiraceae NK4A136* (↑MedScore), ↑SCFA-producers (*Lachnospira*, *Ruminococcaceae* UCG-003); IG-specific: ↓*Lachnospiraceae* genera (*Roseburia*, *Dorea*), ↑B/F ratio. PICRUSt2: IG ↓fermentation/energy pathways, ↑carbohydrate/nucleotide biosynthesis.	IG: weight loss, ↓ BMI, ↓ waist, ↓ triglycerides, ↓ glucose, ↓ HbA1c, ↑ HDL, ↑ physical activity; CG: minimal weight change (− 0.2 kg).	Among MetS adults, energy-restricted MedDiet + PA induced greater weight loss and cardiometabolic improvements vs. *ad libitum* MedDiet, with distinct microbiota signatures: IG showed ↓Firmicutes SCFA-producers (*Haemophilus*, *E. hallii*) + ↑B/F despite no diversity change; *Lachnospiraceae* NK4A136 tracked MedScore across groups; associations suggest BCAA and bile acid pathways may mediate glucose/lipid benefits of energy restriction.
Galié et al., [56]	Crossover RCT, adults with MetS	*N* = 44 (with metabolomic data)	MedDiet vs. non-MedDiet + nuts (50 g/day) (2 months + 1-month washout)	16 S rRNA gene sequencing	No significant gut microbiota changes between interventions; 65 plasma metabolites changed with MedDiet (lipids, acylcarnitines, AA, steroids, TCA intermediates); network analysis: 2 bacterial genera clusters with opposite responses to PC species, ChoE (20:5), TGs, medium/long-chain acylcarnitines.	MedDiet: ↓glucose, ↓insulin, ↓HOMA-IR; nuts alone showed no metabolic improvements.	MedDiet (but not isolated nuts) produced a specific plasma metabolomic signature (65 metabolites) linked to improvements in insulin sensitivity on MetS; no changes in microbiota composition but microbe-metabolite networks suggest interplay between diet → plasma metabolome → cardiometabolic benefits, independent of fecal microbiota shifts.
Galié et al., [57]	Crossover RCT, adults with MetS	*N* = 38 (with gut microbiota data)	MedDiet vs. non-MedDiet + nuts (50 g/day) (2 months + 1-month washout)	16 S rRNA gene sequencing	MedDiet > nuts: ↑ *Lachnospiraceae NK4A136* (inversely associated with insulin, HOMA-IR), ↑ *Ruminococcaceae* (uncultured). Fecal metabolites: ↑cholate (bile acid), ↓cadaverine; *Lachnospiraceae NK4A136* changes correlated with bile acid metabolism and cadaverine shifts explaining insulin improvements.	MedDiet: ↓glucose, ↓insulin, ↓HOMA-IR; nuts alone: no cardiometabolic improvements despite identical nut dose.	Companion paper to Clin Nutr 2021 (same crossover RCT): The MedDiet context (not isolated nuts) specifically enriched *Lachnospiraceae* NK4A136 + shifted fecal bile acids/cadaverine, directly mediating improvements in insulin sensitivity on MetS; demonstrates that whole-diet synergy is required for microbiota-mediated cardiometabolic benefits
Pagliai et al., [58]	Crossover RCT, overweight omnivores with low-to-moderate CVD risk	*N* = 23	Low-calorie MedDiet (MD) vs. vegetarian diet (VD) (3 months)	16 S rRNA gene sequencing	No α/β-diversity changes at family level; MD-specific: ↑ *Enterorhabdus*, ↑ *Lachnoclostridium*, ↑ *Parabacteroides*; VD-specific: ↑ *Anaerostipes*, ↑ *Streptococcus*, ↑ *Clostridium sensu stricto*, ↑ *Odoribacter.* SCFA: MD + 10% propionate vs. VD −28% propionate; MD SCFA changes negatively correlated with inflammatory cytokines (VEGF, MCP-1, IL-17, IP-10, IL-12).	Both diets: weight loss, BMI reduction; MD diet: ↓inflammatory cytokines (VEGF, MCP-1, IL-17).	A 3-month low-calorie MD vs. VD crossover RCT: no major diversity shifts but diet-specific genus changes; MD uniquely increased propionate (+ 10%) with negative SCFA-inflammatory cytokine correlations absent in VD, supporting MD’s superior anti-inflammatory effects via microbiota-SCFA axis despite equivalent weight loss.
Pellegrini et al., [59]	Randomized open-label pilot trial, overweight breast cancer survivors	*N* = 34	MedDiet + probiotics (2 months of probiotics, then 2 months MedDiet alone) vs. MedDiet alone (4 months)	16 S rRNA gene sequencing (baseline and 2-month)	MedDiet+probiotics (2mo): ↑ bacterial species richness, ↑ Chao1 diversity, ↓ B/F ratio; MedDiet alone: no significant microbiota changes; probiotic effects not sustained at 4 months (post-probiotic washout).	Both groups at T4 (4mo): ↓ weight, ↓ BMI, ↓ fasting glucose, ↓ HOMA-IR; MD+probiotics: ↓ waist circumference, ↓ waist-to-hip ratio, ↓ fasting insulin.	Pilot RCT in overweight BC survivors: 2-month MD+probiotics rapidly increased microbiota diversity/richness and ↓B/F ratio (absent with MD alone), with greater improvements in anthropometric and insulin-related outcomes; probiotic effects not sustained after discontinuation, suggesting continuous supplementation may be needed for lasting microbiota benefits.
Pastor-Ibáñez et al., [60]	RCT, HIV-infected individuals on successful antiretroviral therapy	*N* = 102	Supplemented MedDiet with 50 g/day EVOO and 30 g/day walnuts (SMD) vs. regular diet (12 weeks)	16 S rRNA gene sequencing	High-adherence SMD: ↑ gut microbiota diversity/richness; ↑ *Bifidobacterium*, ↑ *Succinivibrio*, ↑ *Butyrivibrio*; ↓ *Bacteroides* (↑ *Prevotella*/*Bacteroides* ratio); *Bifidobacterium*/*Succinivibrio* correlated with ↑ Treg cells, ↓ immune activation.	High-adherence SMD: ↓ total cholesterol, ↓ β-lipoprotein; ↓ immune activation (↓ CD4+/CD8 + HLADR+CD38 + cells); ↓ IFN-γ-producing T-cells; ↑ Treg function.	First HIV-specific MedDiet RCT: 12-week SMD (EVOO+walnuts) in ART-treated HIV+ adults produced dose-dependent microbiota improvements (↑ diversity, ↑ *Bifidobacterium*, ↑ P/B ratio) scaling with adherence; microbiota changes correlated with lipid improvements and immune restoration (↓ activation, ↑ Treg), suggesting SMD restores gut-immune axis disrupted by HIV.
Choo et al., [61]	Crossover RCT, adults with a systolic blood pressure ≥ 120 mmHg and risk factors for cardiovascular disease	*N* = 34	MedDiet + dairy foods (MedDairy) vs. low-fat control diet (LFD) ((8 weeks + 8-week washout)	16 S rRNA gene sequencing	No change in overall community structure or diversity between diets (α/β-diversity); MedDairy-specific taxonomic shifts: ↑*Butyricicoccus* (butyrate producer), ↓*Collinsella*, ↓*Veillonella.*	↑*Butyricicoccus* correlated inversely with systolic BP but positively with fasting glucose during MedDairy; no associations with anthropometry or cognition for other taxa.	In Australian adults at CVD risk, 8-week MedDiet enriched with dairy (MedDairy) did not alter global microbiome diversity vs. LFD but selectively increased butyrate-producing *Butyricicoccus* and decreased *Collinsella/Veillonella*, with *Butyricicoccus* changes linking the diet to lower systolic BP but slightly higher fasting glucose, highlighting nuanced microbiota–cardiovascular interactions of a calcium-adequate MedDiet variant.
Vitale et al., [62]	RCT, overweight/obese adults	*N* = 29	MedDiet vs. habitual Western diet (8 weeks)	16 S rRNA gene sequencing	MedDiet: ↑α-diversity (Shannon), ↑ fecal butyrate (+ 30–40%), ↑postprandial plasma butyrate IAUC; ↑SCFA-producers (*Intestinimonas butyriciproducens*, *Akkermansia muciniphila* ↑), ↓*Ruminococcus torques*, ↓*Coprococcus comes*, ↓*Streptococcus gallolyticus*; butyrate correlated with *Bacteroides xylanisolvens/Roseburia hominis*↑; Control: no microbiota/SCFA changes.	Acute Mediterranean meal: ↓postprandial glucose, ↓insulin (all time points/IAUC); 8-week MedDiet: ↓glucose IAUC, ↓insulin IAUC, ↑OGIS insulin sensitivity; ↓LDL-C; butyrate IAUC inversely correlated with insulin IAUC, positively with OGIS.	MedDiet rapidly improves postprandial glucose/insulin and insulin sensitivity via ↑gut microbiota diversity and ↑ butyrate; butyrate modulates metabolic benefits (correlations with insulin/OGIS); ↓pro-inflammatory taxa (*Ruminococcus torques*) reinforce MedDiet-microbiota-SCFA axis for cardiometabolic protection.
Tagliamonte et al., [63]	RCT, overweight/obese adults	*N* = 82	MedDiet vs. habitual western diet (8 weeks)	Shotgun metagenomics	MedDiet: ↑*A. muciniphila*; ↓OEA/AEA ratio, ↓OEA/PEA ratio, ↓AEA in high-adherence subgroup; baseline EC tone predicted response: low baseline AEA → ↑insulin sensitivity; high OEA/PEA + low AEA → ↑*A. muciniphila*.	High-adherence MedDiet: ↓total cholesterol, ↓insulin resistance (HOMA-IR), ↓hs-CRP (inflammation).	Companion to Meslier et al. Gut 2020: 8-week isocaloric MedDiet modulated endocannabinoidome (↓OEA/AEA, ↓OEA/PEA ratios, ↓AEA) and ↑*A. muciniphila* independently of weight loss; baseline EC tone and microbiome predicted personalized cardiometabolic improvements (insulin sensitivity, cholesterol, inflammation).
Shoer et al., [64]	RCT, adults with prediabetes	*N* = 200	Personalized postprandial-targeting (PPT) diet vs. MedDiet (6 months)	Shotgun metagenomics	PPT > MedDiet changes: 166 of 2,803 features were significant (vs. 49 MedDiet); PPT: ↑gut richness (+ 11 species), ↑diversity, ↑19 gut species (7 *Ruminococcaceae*), ↑9 butyrate compounds, ↑β-mannan degradation; MED: ↑5 gut species (2 *F. prausnitzii* subtypes), ↑bilirubin pathway; mediation: 4 gut species mediate diet→glycemia (*F. prausnitzii SGB_15342*: proteins→time > 140); gut microbiome explains 12.25% of serum metabolite variance.	PPT superior: ↓time > 140 (− 1.9 h/day), ↓HbA1c (− 0.4%), ↓OGTT; ↑SCF, TRAIL (PPT), ↑SIRT2, AXIN1 (MedDiet); microbiome mediates diet→glycemic/metabolic/immune effects.	Companion study to Ben-Yacov et al., Gut 2023: PPT produces three times more molecular changes than MedDiet; strain-level mediation (oral A. naeslundii: drinks→time > 140); 12.25% of metabolite variance was explained by gut microbiome changes; *F. prausnitzii* subtypes show diet-specific effects.
Ben-Yacov et al., [65]	RCT, adults with prediabetes	*N* = 200	Personalized postprandial-targeting (PPT) diet vs. MedDiet (6 months)	Shotgun metagenomics	PPT > MedDiet: ↑ α-diversity (*p* = 0.007 vs. *p* = 0.18), ↑ richness; PPT: 69 species changed (vs. 33 MedDiet); 9 species mediate diet→clinical effects (*UBA11471 sp000434215* mediates MedOil and fat→HbA1c 38%; *Negativibacillus sp000435195* mediates PPT-score→HDL-C 13%).	PPT was superior: ↓HbA1c, ↑HDL-C, ↓triglycerides, ↓time > 140 mg/dL, ↓BMI, ↓BMR; microbiome mediates 13–38% of improvements	PPT outperformed MedDiet with respect to microbiome diversity (↑α-diversity/richness) and cardiometabolic outcomes in prediabetes; causal mediation showing 9 species link dietary changes→clinical benefits; ML models predict personalized responses (baseline microbiome + diet changes).
Wang et al., [68](HPFS subcohort, MLVS)	Prospective cohort, healthy men	*N* = 307	MedDiet score (0–9) long-term adherence (cumulative average from up to 9 FFQs over 26 years)	Shotgun metagenomics	MedDiet ↑microbial plant polysaccharide degradation, ↑SCFA production, ↑pectin metabolism, ↑*F. prausnitzii*, ↑*B. cellulosilyticus* (fiber degraders); no direct association with *P. copri* abundance but MedDiet × *P. copri* interaction: protective association strongest in low-*P. copri* carriers.	MedDiet ↓ composite cardiometabolic risk score (dyslipidemia, hyperglycemia, inflammation biomarkers); microbiome-stratified: stronger protection in low-*P. copri* carriers.	Long-term MedDiet protects against cardiometabolic disease risk via the microbiome (↑fiber degradation/SCFA pathways); microbiome modifies the association; *P. copri* depletion amplifies MedDiet protection (two-three times stronger).
De Filippis et al., [66]	Cross-sectional, healthy Italian adults	*N* = 153	MedDiet adherence (0–11) (7-DDRs) within omnivores, vegetarians, vegans	16 S rRNA gene sequencing	High MedDiet: ↑*Prevotella*, ↑*Lachnospira*, ↑*Xylanisolvent Bacteroides*; ↑fecal SCFA (acetate/propionate/butyrate); ↑polysaccharide degradation genes; ↑microbial gene richness; low adherence: ↑urinary TMAO; SCFA levels in omnivores with high MedDiet adherence were comparable to those observed in vegans; SCFA levels correlated with fiber/plant intake.	High MedDiet adherence: ↑insulin sensitivity, ↓systemic inflammation.	MedDiet adherence (even among omnivores) produces vegetarian-like microbiome (↑*Prevotella*/SCFA pathways) + ↓TMAO; 30% of Western omnivores achieve optimal plant-based microbiota signatures via high plant food consumption.
Peters et al., [67]	Cross-sectional, US Hispanic/Latino adults	*N* = 2,444	Three healthy dietary patterns-the alternate Mediterranean diet (aMED), the Healthy Eating Index (HEI)−2015, and the healthful plant-based diet index (hPDI) (2 × 24-hour recalls)	Shotgun metagenomics	↑ Species enriched in healthier diets were primarily from the class *Clostridia ([Eubacterium] eligens*,* Butyrivibrio crossotus*, and *Lachnospiraceae bacterium TF01-11);* ↓ *Acidaminococcus intestine*; aMED associated with pyruvate: ferredoxin oxidoreductase; hPDI associated with L-arabinose/lactose transport; poorer diet quality associated with manganese/iron transport, adhesin protein transport, and nitrate reduction pathways.	↓ Fasting insulin, HOMA-IR, and waist-to-hip ratio; healthy diet–enriched *Clostridia* species (e.g., *Ruminococcus lactaris*, *Lachnospiraceae bacterium TF01-11*, *Butyrivibrio crossotus*) were associated with ↓ triglycerides, ↓ insulin resistance, and ↓ adiposity measures; a microbiome score combining species associated with ≥ 2 dietary patterns was associated with ↓ fasting insulin, HOMA-IR, and waist-to-hip ratio, certain healthy diet–enriched species were linked to lower HDL cholesterol; poorer diet–related *Flavonifractor plautii* was associated with favorable glycemic and adiposity traits.	Healthy dietary patterns in US Hispanic/Latino adults are consistently associated with enrichment of fiber-fermenting *Clostridia* species. Intakes of whole grains, fruit, and vegetables are primary drivers. Some diet-related species and functions correlate with favorable cardiometabolic traits, but unexpected associations (e.g., *Flavonifractor plautii* linked to better glycemic traits despite being related to poorer diet) highlight complexity and the need for diverse population replication.

7-DDR, 7-day dietary record; AA, amino acids; AEA, anandamide; aMED, alternate Mediterranean diet score; ART, antiretroviral therapy; B/F ratio, Bacteroidetes-to-Firmicutes ratio; BC, breast cancer; BCAA, branched-chain amino acid; BMI, body mass index; BMR, basal metabolic rate; BP, blood pressure; CG, control group; ChoE, cholesteryl ester; CRP, C-reactive protein; CVD, cardiovascular disease; EC, endocannabinoid; EVOO, extra-virgin olive oil; FFQ, food-frequency questionnaire; GGT, gamma-glutamyl transferase; HbA1c, glycated hemoglobin; HDG, healthy dietary guidelines; HDL-C, high-density lipoprotein cholesterol; HEI, Healthy Eating Index; HIV, human immunodeficiency virus; HOMA-IR, homeostatic model assessment of insulin resistance; hPDI, healthful plant-based diet index; hs-CRP, high-sensitivity C-reactive protein; IAUC, incremental area under the curve; IFN-γ, interferon gamma; IG, intervention group; IL, interleukin; IP-10, interferon gamma-induced protein 10; LDL-C, low-density lipoprotein cholesterol; LFD, low-fat diet; MCP-1, monocyte chemoattractant protein-1; MD, Mediterranean diet; MedDiet, Mediterranean diet; MedDairy, Mediterranean diet supplemented with dairy foods; MetS, metabolic syndrome; ML, machine learning; MLVS, Men’s Lifestyle Validation Study; OEA, oleoylethanolamide; OGTT, oral glucose tolerance test; OGIS, oral glucose insulin sensitivity index; PA, physical activity; P/B ratio, Prevotella-to-Bacteroides ratio; PC, phosphatidylcholine; PEA, palmitoylethanolamide; PICRUSt2, Phylogenetic Investigation of Communities by Reconstruction of Unobserved States 2; PPT, personalized postprandial-targeting diet; RCT, randomized controlled trial; rRNA, ribosomal RNA; SCF, stem cell factor; SCFA, short-chain fatty acid; SGB, species-level genome bin; SMD, supplemented Mediterranean diet; TCA, tricarboxylic acid; TC, total cholesterol; TG, triglyceride; TMAO, trimethylamine-N-oxide; TRAIL, tumor necrosis factor-related apoptosis-inducing ligand; Treg, regulatory T cell; VD, vegetarian diet; VEGF, vascular endothelial growth factor; ↑, increased; ↓, decreased

#### Multiomic Integration to Identify Mechanisms, Causal Models, and Functional Biomarkers

Metabolomics adds a functional layer that cannot be captured by microbial taxonomy alone. While 16 S rRNA gene sequencing and shotgun metagenomics describe microbial composition or genetic potential, metabolomics provides a closer readout of biochemical activity at the host–microbial interface. In MedDiet research, this is particularly relevant because many key mechanisms are defined not by the presence of a single taxon but by the production, depletion, or transformation of metabolites related to fiber fermentation, bile acid remodeling, polyphenol biotransformation, amino acid metabolism, lipid metabolism, and inflammatory signaling [53, 56, 57, 68, 72, 73].

The integration of microbiome and metabolome data can help distinguish common from pathway-specific mechanisms. Common mechanisms are reproducible across MedDiet interventions, such as shifts toward saccharolytic fermentation and lower proteolytic metabolism. Pathway-specific mechanisms include TMAO and PAGln-related atherothrombotic pathways, bile acid signaling, phenolic metabolite production, cholesterol biotransformation, acylcarnitine metabolism, ceramide and sphingolipid networks, and endocannabinoid-related signatures. Multiomic integration thus links dietary exposures, microbial genes or taxa, circulating or fecal metabolites, and cardiometabolic phenotypes within the same analytical framework [53, 56, 57, 69].

Methodologically, several approaches can be used to move beyond descriptive associations. Mediation analyses can estimate whether microbiome or metabolomic features statistically explain part of the association between MedDiet adherence and cardiometabolic outcomes. Longitudinal models with repeated measures can help clarify temporal ordering between dietary change, microbial remodeling, metabolite variation, and clinical response. Network-based approaches can identify co-varying modules of foods, microbial functions, metabolites, and biomarkers, while sparse canonical correlation and multi-block methods can detect shared structures across high-dimensional omic datasets. Machine-learning models may help identify predictive signatures of response, but they require external validation, careful control of overfitting, and testing across populations with different baseline diets, medication use, and cardiometabolic profiles [74]. Crucially, most current multiomic evidence is observational and cross-sectional; without repeated measures or interventional designs, these methods can describe covariation but cannot establish the direction of causation.

A key objective of this methodological shift is the development of functional biomarkers that link discovery to clinical use. These biomarkers should not consist of single bacterial taxa but reproducible panels combining microbial functional capacity, metabolite profiles, and host clinical features. Candidate biomarker domains include microbial gene richness, plant polysaccharide degradation, SCFA-related activity, bile acid transformation, polyphenol-derived metabolites, TMA/PAGln-related pathways, lipid intermediates, inflammatory markers, and microbial cholesterol metabolism [23, 31, 32, 38, 53]. Such panels may improve dietary adherence assessment, identify biological responders and non-responders, and guide future microbiome-informed MedDiet interventions. However, their clinical use will require prospective validation, standardized analytical pipelines, harmonized metabolite measurement, and evidence that biomarker-guided counseling improves outcomes beyond conventional dietary advice.

### Translational and Precision Nutrition Perspectives 

#### Microbiome-Based Biomarkers and Personalized Mediterranean Diet Response

The translational challenge is to determine whether microbiome- and metabolome-derived signatures can improve dietary guidance beyond conventional dietary scores and cardiovascular risk factors. While Sect. 3.3 outlines the analytical approaches needed to identify functional biomarkers, clinical translation requires an additional step: demonstrating that these biomarkers improve prediction, adherence, or cardiometabolic outcomes in real-world settings. Current prediction models remain largely focused on surrogate outcomes, including postprandial glycemia, triglyceride responses, insulin resistance, adiposity, and inflammatory markers, rather than hard cardiovascular endpoints such as myocardial infarction, stroke, or cardiovascular mortality. Microbiome-informed MedDiet counseling should therefore be viewed as a promising refinement of standard dietary advice rather than an established clinical intervention [21, 75–77].

At present, these findings are insufficient to support routine microbiome profiling or microbiome-guided dietary prescription in cardiovascular practice. A change in clinical practice would require additional evidence at several levels: reproducible and externally validated microbial or multiomic biomarkers; standardized analytical procedures and clinically interpretable thresholds; demonstration that these biomarkers provide incremental predictive or therapeutic value beyond established cardiovascular risk factors and conventional dietary assessment; and randomized or pragmatic trials showing that microbiome-informed Mediterranean diet counseling improves clinically meaningful cardiometabolic or cardiovascular outcomes compared with standard evidence-based dietary counseling. Feasibility, cost-effectiveness, and reproducibility across populations would also need to be established before implementation in routine care. Thus, current microbiome-derived information should be considered a promising tool for future precision nutrition rather than a clinically validated basis for modifying established cardiovascular prevention strategies.

#### Microbiome-Informed Mediterranean Diet Counseling: From Biomarkers to Implementation

Microbiome-informed MedDiet counseling should be framed as a refinement of evidence-based dietary guidance rather than a replacement for it. The objective is not to prescribe completely different dietary patterns but to optimize established MedDiet principles according to an individual’s microbial functional profile and metabolic phenotype. In practice, personalization may involve adapting fiber type and dose, legumes and whole-grain choices, polyphenol-rich foods, fermented foods, nut intake, and animal protein intake according to baseline microbiome configuration, metabolomic profile, cardiometabolic phenotype, gastrointestinal tolerance, medication use, and predicted postprandial metabolic responses [53, 66, 67, 69, 72, 78, 79]. The goal is not simply to increase α-diversity but to promote reproducible functional shifts: greater saccharolytic fermentation, enhanced polyphenol biotransformation, favorable bile acid signaling, improved gut barrier integrity, lower TMA-producing capacity, and reduced systemic inflammation [21, 53, 56, 57, 69, 74, 75].

Translation will require pragmatic clinical trials comparing standard MedDiet advice with microbiome-informed MedDiet counseling, using clinically meaningful endpoints, cost-effectiveness analyses, and implementation strategies feasible in routine care. Until then, microbiome-informed personalization should be framed as a refinement of evidence-based dietary guidance and adherence support, not as a stand-alone therapeutic substitute [64, 65, 76, 77, 80].

#### Inter-Individual Variability in Response to the Mediterranean Diet

One of the most important implications of microbiome research is the recognition that individuals do not respond uniformly to the MedDiet. Inter-individual variability appears to result from complex interactions among dietary adherence, baseline microbial functional capacity, host metabolism, medication exposure, genetic background, and cardiometabolic phenotype. Individuals with higher baseline capacity for plant polysaccharide degradation, SCFA production, microbial polyphenol biotransformation, bile acid remodeling, or cholesterol metabolism may be more able to convert MedDiet substrates into favorable changes in insulin sensitivity, lipid metabolism, inflammatory tone, and vascular risk [53, 57, 63, 67]. Conversely, lower fermentative capacity, greater reliance on proteolytic metabolism, dysbiosis-associated taxa, impaired gut-barrier function, or adverse metabolic profiles may attenuate or delay these beneficial responses. Importantly, randomized trials have shown that cardiometabolic improvements can occur without major changes in α- or β-diversity, indicating that responder status should be inferred from functional microbial and metabolomic readouts rather than diversity metrics alone [55, 56, 58, 61]. Bile acid metabolism may be especially informative for precision nutrition because the cardiometabolic relevance of fecal bile acids appears to depend on the abundance of microbial bile-acid metabolizing enzymes, suggesting that the same dietary exposure may have different consequences for lipid metabolism and adiposity depending on microbial functional capacity [81]. Recent large-scale metagenomic studies further indicate that diet-responsive microbial signatures are not limited to traditional genus-level markers but involve species- and function-level microbial patterns associated with habitual diet, cardiometabolic health, and response to dietary interventions. This supports the need for externally validated microbial signatures rather than single-taxon biomarkers, especially when translating MedDiet–microbiome findings across populations [79].

## Limitations

 Several limitations should be considered when interpreting this review and the underlying evidence. First, this article is a narrative review rather than a systematic review or meta-analysis; therefore, although the literature was identified through a targeted and transparent search strategy, study selection and qualitative synthesis may remain susceptible to selection and interpretation bias, and no formal risk-of-bias assessment or quantitative evidence synthesis was performed. Second, the available literature is highly heterogeneous with respect to study populations, definitions and assessment of MedDiet adherence, intervention duration, sample size, biospecimen collection, sequencing technologies, taxonomic resolution, metabolomics platforms, and statistical and bioinformatic pipelines, which limits direct comparability across studies.

 Third, many human studies remain relatively small and predominantly evaluate surrogate cardiometabolic outcomes, including adiposity, insulin resistance, lipid profiles, inflammatory markers, and blood pressure, rather than incident myocardial infarction, stroke, cardiovascular mortality, or other hard cardiovascular endpoints. Fourth, stool microbiome composition and fecal metabolite concentrations do not necessarily reflect microbial functional activity or systemic exposure, while several metabolites discussed in this review, including TMAO and bile acids, are jointly influenced by microbial and host metabolism. Fifth, medication use, renal function, baseline dietary patterns, adiposity, age, geography, and cardiometabolic phenotype may confound or modify observed associations and contribute to between-study heterogeneity. Finally, cross-sectional associations, mediation analyses, and multiomic correlations cannot by themselves establish biological causality, particularly when temporal ordering between dietary exposure, microbiome remodeling, metabolite changes, and cardiovascular outcomes has not been demonstrated.

### Knowledge Gaps

 Despite substantial progress, several important knowledge gaps remain. First, direct evidence linking MedDiet–induced microbiome remodeling to incident cardiovascular events and cardiovascular mortality remains limited, as most human studies have focused on intermediate cardiometabolic phenotypes. Consequently, it remains uncertain which microbial functions or metabolites are causal mediators of cardiovascular benefit, which act primarily as effect modifiers of dietary response, and which are biomarkers of dietary exposure or host metabolic status without a direct causal role.

 Second, there is currently no consensus regarding the optimal microbial, metabolomic, or integrated multiomic signatures for characterizing response to the MedDiet. Few candidate biomarkers have been externally validated across independent populations, and their temporal stability, reproducibility across analytical platforms, and clinically meaningful thresholds remain uncertain. It is also unclear whether microbiome-derived information provides sufficient incremental predictive or therapeutic value beyond established cardiovascular risk factors, dietary assessment, and conventional biomarkers to justify its use in routine clinical practice.

 Additional uncertainty concerns the generalizability of current findings across populations with different geographic, ethnic, dietary, and cardiometabolic characteristics. The extent to which medications, renal function, baseline diet, host genetics, adiposity, age, and other environmental or clinical factors modify MedDiet–microbiome interactions also remains incompletely characterized. These unresolved issues currently constrain both causal inference and the translation of microbiome-related findings into clinically actionable precision-nutrition strategies.

### Future Directions

 Future research should prioritize longitudinal studies incorporating repeated assessments of dietary intake, gut microbiome composition and function, metabolomic profiles, and cardiometabolic phenotypes to establish the temporal sequence linking MedDiet exposure, microbial remodeling, metabolite changes, and host response. Randomized controlled trials should incorporate prespecified microbiome and metabolomic outcomes and, when feasible, evaluate hard cardiovascular endpoints or clinically validated intermediate outcomes rather than relying predominantly on cross-sectional associations or surrogate biomarkers.

 Analytical approaches should also move beyond descriptive associations to distinguish potential causal mediators, effect modifiers, and predictive biomarkers. Causal mediation analyses, diet-by-microbiome interaction models, repeated-measures analyses, and mechanistically informed experimental studies may help clarify whether specific microbial functions or metabolites contribute directly to the cardiometabolic effects of the MedDiet or primarily identify individuals with different response profiles. Candidate microbial and multiomic signatures should subsequently undergo external validation across independent populations, geographic settings, and analytical platforms before being considered for clinical use.

 From a translational perspective, pragmatic trials should compare standard evidence-based MedDiet counseling with microbiome-informed approaches to determine whether personalization provides additional benefits in dietary adherence, cardiometabolic risk profiles, cardiovascular outcomes, or other clinically meaningful endpoints. Such studies should also assess feasibility, cost-effectiveness, accessibility, and reproducibility in routine care. Finally, harmonization of biospecimen collection, sequencing and metabolomics procedures, bioinformatic pipelines, statistical analyses, and reporting standards will be essential to improve comparability across studies and support the development of clinically interpretable microbiome-informed precision-nutrition strategies.

## Conclusion

Current evidence supports a role for the gut microbiome in shaping the cardiometabolic response to the MedDiet, particularly through changes in microbial functions and metabolite pathways. However, the heterogeneity of microbial responses and the predominance of intermediate cardiometabolic outcomes limit the strength of causal inference.

However, the available evidence does not establish that microbiome remodeling is a necessary or sufficient causal mediator of the cardiovascular benefits of the MedDiet. Most studies have evaluated intermediate cardiometabolic outcomes, and direct evidence linking diet-induced microbiome changes to incident myocardial infarction, stroke, cardiovascular mortality, or other hard cardiovascular endpoints remains limited. Therefore, while the MedDiet itself is supported as an evidence-based dietary strategy for cardiovascular prevention, current evidence is insufficient to support routine microbiome profiling, measurement of microbiome-derived metabolites, or microbiome-guided dietary prescription in clinical practice. Microbiome and multiomic information should therefore currently be regarded as a promising approach for understanding inter-individual variation in MedDiet response and for developing future precision-nutrition strategies rather than as an established clinical tool.

## Key References

- Fromentin S, Forslund SK, Chechi K, et al. Microbiome and metabolome features of the cardiometabolic disease spectrum. Nat Med. 2022, 28(2):303–314. [19]
  - ○ Large multiomic study that maps microbiome and metabolome signatures across the cardiometabolic disease spectrum and disentangles disease- from treatment-related features, reinforcing the shift from taxonomy to function and the confounding influence of medication.
- Valles-Colomer M, Menni C, Berry SE, Valdes AM, Spector TD, Segata N. Cardiometabolic health, diet and the gut microbiome: a meta-omics perspective. Nat Med. 2023, 29(3):551–561. [75]
  - ○ Authoritative synthesis that frames diet-microbiome-cardiometabolic interactions through integrated meta-omics and articulates why species- and function-level, externally validated signatures are required for precision nutrition.
- García-Gavilán JF, Atzeni A, Babio N, et al. Effect of 1-year lifestyle intervention with energy-reduced Mediterranean diet and physical activity promotion on the gut metabolome and microbiota: a randomized clinical trial. Am J Clin Nutr. 2024, 119(5):1143–1154. [67]
  - ○ PREDIMED-Plus randomized trial linking a 1-year energy-reduced Mediterranean-diet intervention to reproducible functional remodeling of the gut metabolome, illustrating diet-induced microbial changes of cardiometabolic relevance.
- Li C, Stražar M, Mohamed AMT, et al. Gut microbiome and metabolome profiling in Framingham heart study reveals cholesterol-metabolizing bacteria. Cell. 2024, 187(8):1834–1852 e1819. [71]
  - ○ Population-scale multiomic profiling that identifies gut bacteria capable of metabolizing cholesterol and links them to lower host cholesterol, exemplifying how function-level discovery can yield mechanistically grounded biomarkers.
- Gao P, Rinott E, Dong D, et al. Gut microbial metabolism of bile acids modifies the effect of Mediterranean diet interventions on cardiometabolic risk in a randomized controlled trial. Gut Microbes. 2024, 16(1):2426610. [78]
  - ○ Trial-based analysis showing that the cardiometabolic response to a Mediterranean diet depends on microbial bile-acid-metabolizing capacity, a concrete example of microbiome-conditioned, responder-specific dietary effects.
- Bermingham KM, Linenberg I, Polidori L, et al. Effects of a personalized nutrition program on cardiometabolic health: a randomized controlled trial. Nat Med. 2024, 30(7):1888–1897. [77]
  - ○ Randomized trial demonstrating that a microbiome-informed personalized nutrition program improves cardiometabolic markers beyond general dietary advice, supporting the translational feasibility of the precision-nutrition strategies discussed here. 

## Data Availability

No datasets were generated or analysed during the current study.
